# Coping Strategies Used by Health Care Workers in Ecuador During the COVID-19 Pandemic: Observational Study to Enhance Resilience and Develop Training Tools

**DOI:** 10.2196/47702

**Published:** 2023-09-06

**Authors:** María Asunción Vicente, Eva Gil Hernández, Irene Carrillo, César Fernández, Adriana López-Pineda, Mercedes Guilabert, Jimmy Martín-Delgado, Carlos Solis, Karla Camba, Wilson Ricardo Cañizares Fuentes, José Joaquín Mira

**Affiliations:** 1 Health Psychology Department Miguel Hernandez University Elche Spain; 2 Foundation for the Promotion of Health and Biomedical Research of Valencia Region Sant Joan d’Alacant Spain; 3 Hospital Luis Vernaza Junta de Beneficencia de Guayaquil Guayaquil Ecuador; 4 Instituto de Investigación e Innovación en Salud Integral Facultad de Ciencias Médicas Universidad Católica de Santiago de Guayaquil Guayaquil Ecuador; 5 Endocrinology Service Hospital IEES Norte Los Ceibos Guayaquil Ecuador; 6 Facultad de Medicina Universidad de Guayaquil Guayaquil Ecuador; 7 Facultad de Ciencias de la Salud Universidad de Especialidades Espíritu Santo Guayaquil Ecuador

**Keywords:** COVID-19, coping, mental health, social support, health professionals, psychological well-being, resilience, coping strategy, used, health care worker, pandemic, well-being, professional, intervention

## Abstract

**Background:**

The COVID-19 pandemic has generated immense health care pressure, forcing critical decisions to be made in a socially alarmed environment. Adverse conditions have led to acute stress reactions, affective pathologies, and psychosomatic reactions among health personnel, which have been exacerbated by the successive waves of the pandemic. The recovery of the entire health system and its professionals has been hindered, making it essential to increase their resilience.

**Objective:**

This study aimed to achieve 2 primary objectives. First, it sought to identify coping strategies, both individual and organizational, used by health care workers in Ecuador to navigate the acute stress during the early waves of the pandemic. Second, it aimed to develop training materials to enhance team leaders' capabilities in effectively managing high-stress situations.

**Methods:**

The study used qualitative research techniques to collect information on institutional and personal coping strategies, as well as consensus-building techniques to develop a multimedia psychological tool that reinforces the resilience of professionals and teams in facing future crises.

**Results:**

The findings from the actions taken by health care workers in Ecuador were categorized into 4 types of coping strategies based on Lazarus' theories on coping strategies. As a result of this study, a new audiovisual tool was created, comprising a series of podcasts, designed to disseminate these strategies globally within the Spanish-speaking world. The tool features testimonials from health care professionals in Ecuador, narrating their experiences under the pressures of providing care during the pandemic, with a particular emphasis on the coping strategies used.

**Conclusions:**

Ensuring the preparedness of health professionals for potential future outbreaks is imperative to maintain quality and patient safety. Interventions such as this one offer valuable insights and generate new tools for health professionals, serving as a case study approach to train leaders and improve the resilience capacity and skills of their teams.

## Introduction

### COVID-19 Pandemic in Ecuador

On March 11, 2020, the World Health Organization (WHO) declared COVID-19 a global pandemic, recognizing its rapid spread and the threat it posed to public health and well-being. In nearly 3 years since the declaration, the pandemic has continued to have a profound impact on the world, with a staggering number of confirmed cases reported globally. As of the latest data, 672,454,287 positive cases of COVID-19 have been recorded, along with 6,849,173 reported deaths. To mitigate the spread of the virus and protect public health, massive efforts have been made to distribute vaccines, with 13,283,899,733 doses having been administered to individuals worldwide (February 9, 2023) [1].

In the Republic of Ecuador, the COVID-19 pandemic has taken a significant toll on the population, with a staggering number of confirmed cases reported to the WHO. Between January 2020 and February 2023, the total number of confirmed cases has surpassed 1 million, with an official death toll of 35,965 individuals (172 health care workers [HCWs] in Guayas, and more than 700 HCWs throughout the country), as of the latest report to the WHO [2]. An examination of the excess death rates during the COVID-19 pandemic reveals that, among the countries in Latin America, Ecuador has experienced one of the highest levels of impact [3]. The 2 provinces most affected with confirmed cases of COVID-19 in Ecuador were Pichincha (percentage relative to the total number of cases: 37%) and Guayas (15%) [4], which are also the most populous provinces in the country. This study was conducted with HCWs in the city of Guayaquil, the capital of the province of Guayas.

### Strategies for the Psychological Support of the Health Care Workforce During the COVID-19 Pandemic

The COVID-19 pandemic has not only affected the economy and public health but also the physical and psychological well-being of HCWs who have worked tirelessly on the front lines. The most commonly reported psychological responses among health care professionals include distress (40%-54%), anxiety (37%-72%), depression (38%-53%), sleep disturbances (8%-72%), and burnout (26%-68%) [5]. This emotional distress was close to the experience perceived in the aftermath of higher stressful situations [6] and has earned health care professionals the title of the “second victims” of the pandemic [7], impacting both their health [8] and the quality of care they provide to patients [9,10]. In this context, it was necessary for health organizations to implement strategies for the psychological support of the health care workforce [11].

In 2021, at the beginning of the pandemic, the European Observatory on Health Systems and Policies published an extensive document with 20 key strategies to improve resilience during the COVID-19 pandemic [12]. The crisis highlights the importance of maintaining an adequate health care workforce, which involves not only adequate numbers of health care professionals but also safeguarding their physical and mental well-being to ensure continued patient care [13]. The European Researchers’ Network Working on Second Victims study of 35 countries revealed that there were common responses across all continents in addressing the challenge posed by the pandemic, and that 24-hour hotline format for psychological support was the most commonly used tool for supporting HCW mental health, with extensive use of self-rescue tools such as apps and websites [14].

Nevertheless, there remains a significant gap in the comprehensive investigation of the coping mechanisms used by frontline health care workers while delivering care to patients with COVID-19. These strategies are crucial for managing acute stress and ensuring their consistent return to work, enabling them to fulfill their responsibilities under challenging circumstances.

### Proposal and Context: “BE + Against COVID-19”

During the first wave of the COVID-19 pandemic in Spain (from March to June 2020), the “BE+ Against COVID-19” platform was launched, consisting of a multilingual web portal and an app with resources and materials to mitigate acute stress among health care and nonhealth care professionals associated with the crisis caused by the pandemic [7]. From this platform, led by JJM and composed of over 50 researchers and HCWs from Spain and Latin America, a battery of 19 multimedia resources was proposed to mitigate the acute stress associated with the crisis situation caused by the high care pressure. These support resources included a self-administered scale for acute stress assessment [15], infographics with tips for coping with the impact of the pandemic on professionals (eg, steps for progressive muscle relaxation and Stop technique) and health care teams (eg, group techniques such as defusing and debriefing), and videos for the guided performance of mindfulness or emergency minute exercises, among others.

Thanks to 2 grants (detailed in the Acknowledgments section), it was possible for the Spanish team of the “BE+ Against COVID-19” platform to collaborate with a group of health care professionals from Ecuador, specifically with the IESS Los Ceibos General Hospital in Guayaquil to bring the proposals of “BE+ Against COVID-19” and also learn from their experiences during the pandemic with the intention of developing new psychological support tools for health professionals and add them to the existing resources of this platform. IESS Los Ceibos General Hospital in Guayaquil served as a sentinel care center for patients with COVID-19 during the months of March and April 2020 when the first wave of the pandemic caused the toughest moments in Guayas province. As in all countries, the sudden onset of the pandemic exposed hospital health personnel to extremely challenging working conditions. The work presented in this study arises from this collaboration.

### Aim

This project had a dual objective. The primary purpose of the study was to identify coping actions and strategies that HCWs in Ecuador used to navigate the challenges posed by the health care emergency. By examining both the institutional and the individual levels of health care personnel, the study aimed to uncover lessons in resilience that can be applied in similar situations in the future. This includes identifying actions and strategies that have had a positive impact and contributed to effective management of the emergency.

Despite being aware of the daunting challenges and inadequate resources they would encounter, these health care professionals exhibited remarkable resilience, continuously recovering and resuming their duties day after day. What factors served as deterrents to surrendering, and how did they discover avenues for recuperation and redirect their focus toward fulfilling their professional obligations?

The secondary aim was to develop a multimedia psychological tool to train leaders of professional groups, enhancing their ability to support their teams and reinforce individual and team resilience.

## Methods

### Study Design

This observational study was conducted in 2 phases. The first phase involved qualitative research techniques to collect information on institutional and personal coping strategies, while the second phase used consensus-building techniques and was focused on developing a multimedia psychological tool to reinforce the resilience of professionals and teams in facing future crises.

### Ethics Approval

The study was approved by the Miguel Hernández University committee for responsible research (AUT.INT.MVR.07.21).

### Participants and Data Collection

The first phase of this study used the focus group technique to capture the experiences, emotions, and measures taken by health care personnel to cope with the emotional consequences of the pandemic. The study made efforts to include primary and hospital care professionals from the city of Guayaquil. Focus groups, consisting of 4 to 9 participants each, were conducted until data saturation was achieved. The snowball technique was used to recruit health care professionals. The study had a singular inclusion criterion: being an HCW affiliated with the IESS Los Ceibos General Hospital and having a substantial work experience during the COVID-19 pandemic.

Throughout each focus group session, 2 adept moderators, 1 from Ecuador and the other from Spain, skillfully guided the conversation and posed pertinent questions to elicit responses from the participants. Textbox 1 displays the script content used in the sessions held with health care groups from the hospital in Guayaquil, Ecuador. This script was divided into 2 information blocks related to psychological well-being aspects at the organizational and individual levels.

**Script used in the focus groups and structured in 2 parts.**

**Organization or institutional level**

O_Q1. What changes have taken place in the organization of the center that have been positive and would not have been applied if not for the COVID-19 pandemic?

O_Q2. What changes have taken place in the center's staff that have been positive and would not have been applied if not for the COVID-19 pandemic?

O_Q3. What changes have taken place in the center's resource and equipment management that have been positive and would not have been applied if not for the COVID-19 pandemic?

O_Q4. From a constructive perspective, how could decisions in crisis situations be improved in the future?

**Individual experience**

I_Q1. What have you done that has worked well for you to feel better and handle the care of patients with COVID-19 during times of greater uncertainty and crisis?

I_Q2. What have you learned from other coworkers that works better for handling the care of patients with COVID-19 during times of greater uncertainty and crisis?

I_Q3. What advice would you give to future professionals (currently in training) in the face of a situation like that experienced with COVID-19?

### Data Analysis

The data gathered during the focus groups were recorded and transcribed verbatim, with full permission and anonymity granted to all participants. The research team then systematically extracted various coping strategies, both at the organizational and individual levels, as narrated by the health care professionals from Ecuador. These strategies will be presented in detail in the Results section. While following the guide outlined in Textbox 1 for the oral interviews, a diverse range of experiences emerged, encompassing both organizational and individual aspects. The individual experiences held greater prominence due to the emotionally charged nature of the sessions, as demonstrated by the findings.

The coping actions listed and described by the Ecuadorian HCWs were categorized into 4 types of coping strategies based on the well-known Lazarus' theories on coping strategies [16,17]. These types of coping strategies are commonly referred to as emotion-focused adaptive coping strategies (ACS), problem-focused ACS, emotion-focused maladaptive coping strategies (MACS), and problem-focused MACS.

First, emotion-focused ACS involve seeking social support and emotional expression to manage feelings and emotions associated with the problem situation in a positive manner. This type of coping strategy is helpful in situations where the individual may not have control over the problem at hand.

Second, problem-focused ACS, on the other hand, involve activities aimed at solving the problem or restructuring thoughts that involve changing the situation or its meaning. This type of coping strategy is helpful in situations where the individual has control over the problem at hand.

Third, emotion-focused MACS involve social isolation and self-blame, which are not helpful in managing the problem situation in a positive manner. Individuals who engage in these types of coping strategies may require additional support and intervention.

Finally, problem-focused MACS involve avoiding the problem or stressful situations and engaging in wishful thinking (fantasizing about alternative realities). While problem-avoidance strategies may be considered adaptive as temporary measures, they are not helpful in the long term and may hinder problem-solving efforts.

By categorizing the actions of the Ecuadorian health care professionals into these 4 types of coping strategies, we can better understand the effectiveness of their responses to the COVID-19 pandemic and develop targeted interventions to improve their resilience in the face of future outbreaks.

### Enhancing Resilience of Individuals and Teams: Training Staff and Developing Psychological Tools

Through in-depth narratives shared by HCWs from Guayaquil, this study identified individual coping strategies and organizational decisions that had a positive impact. Key elements were defined for group leaders to implement in order to enhance their teams' resilience. A methodology for training team leaders was also determined, and case study materials were developed as a result of these findings. Subsequently, a novel multimedia psychological support tool in the form of a podcast series was created. The podcast scripts were collaboratively crafted by a team of psychologists and multimedia engineers, ensuring well-designed and engaging content. The recording process involved skilled actors and actresses to bring the podcasts to life. The development protocol for the podcast series is presented in Textbox 2. To ensure confidentiality, fictitious names and professions were used in the podcast scripts.

These podcasts aim to compile the experiences and challenges encountered by health care professionals during the pandemic, with a specific focus on the coping strategies used in Ecuador. The ultimate goal is to offer support and guidance to future professionals and teams who may face similar high-pressure health care situations.

**Six-step podcast development protocol used in this study.**

**Podcast development protocol**

1. Define objectives: outline podcast goals—coping strategies, personal experiences, and emotional support. Identify desired outcomes—resilience, well-being, and coping skills.
2. Content planning: develop relevant topics, subtopics, and key messages aligned with objectives and audience needs.
3. Plan and record: determine episode format, prepare scripts, and conduct high-quality recordings.
4. Edit and produce: ensure audio quality, coherence, and appropriate length.
5. Create artwork and branding: design visually appealing cover art and maintain consistent branding.
6. Publish and distribute: host on platforms, submit to directories, and share through relevant channels.

## Results

### Overview

The group sessions were held on January 24 and 26, 2022. A total of 37 health care professionals, comprising 23 females and 14 males, participated in 6 separate focus groups. The health care professionals and administrative workers were affiliated with the IESS Los Ceibos General Hospital and included social workers, psychology and psychiatry staff, occupational risk management staff, heads of service from different specialties, nurses, general medicine staff, pediatric and neonatology staff, human resources, planning, and communication staff from the hospital, critical care general practitioners, epidemiologists, general practitioners, and nutritionists.

This work has developed its results in 2 phases too. In the first phase of analysis, exploration, obtaining and classifying coping strategies for HCWs, and the second phase of elaboration of recommendations in multimedia digital format. Each of these phases are described in depth in the following subsections.

### Results 1: Coping Strategies Classification

Researchers EGH and IC collaborated in analyzing the transcribed text from the group sessions and successfully identified and extracted over 70 distinct coping strategies, comprising both organizational and individual approaches. The complete list and its classification were supervised by researchers and HCWs (JJM, JMD, CS, KC, WRCF, and ALP) who actively participated in the focus group sessions. The resulting classification is presented in Textboxes 3 and 4.

**Adaptive coping strategies (ACS).**

**Emotion-focused ACS**

- **Individual**
  Acquire and comprehend the intricacies of life.
  Allow yourself time to relax and reflect.
  Acknowledge and express your fears.
  Religious faith or cultivate a strong belief system.
  Create videos with uplifting messages.
  Seek professional counseling or therapy.
  Maintain a calm demeanor.
  Practice mindfulness and meditation techniques.
  Regain mobility and a sense of freedom.
- **Organization level**
  Social support from coworkers or family members: engaging in conversation about the issue, making video calls, receiving positive messages and messages of concern from coworkers or family members.
  Support groups: joining a group of individuals facing similar challenges to receive emotional and social support.
  Empathy: showing understanding and concern for the experiences and feelings of others.
  Provision of food and beverages: providing food and beverages as a potential reinforcer for staff.
  Active listening: learning how to effectively listen to colleagues and provide support.

**Problem-focused ACS**

- **Individual**
  Understanding the situation.
  Keeping a daily report.
  Verifying official information.
  Making decisions logically rather than emotionally.
  Assessing risks and ensuring safety.
  Contributing to the community through solidarity.
  Adhering to protective measures.
  Taking breaks before work.
  Performing various duties within the hospital.
  Using telemedicine.
  Minimizing patient distress during emergency situations.
  Assisting patients in contacting their families.
  Possessing a strong sense of vocation for the job.
  Maintaining a positive outlook.
  Receiving mental health support through phone calls.
- **Organization level**
  Teamwork.
  Fellowship.
  Team spirit.
  Communication: group chats.
  Exchange of feedback among colleagues.

**Maladaptive coping strategies (MACS).**

**Emotion-focused MACS**

- Isolation

**Problem-focused MACS**

- Avoid focusing on the number of deceased individuals.
- Avoiding exposure to media: do not access social networks.
- Diverting attention: engage in conversation about other topics unrelated to the pandemic.
- Limit exposure to news related to the pandemic.
- Create a reality separate from external events.
- Engaging in risky or extreme activities: participate in activities such as skydiving, diving, tattooing, and hair dyeing.
- Pursuing hobbies or pleasurable activities: engage in activities such as singing, cooking or baking, listening to music, attending painting classes, reading, using TikTok, and watching television or movies or series.
- Physical exercise: engage in sports or physical activity.

Textboxes 3 and 4 provide a comprehensive overview of the coping strategies implemented by the professionals. Specifically, Textbox 3 outlines the list of ACS, while Textbox 4 displays the list of MACS. These textboxes offer valuable insights into the coping mechanisms used by HCWs during times of crisis and can inform future interventions and support strategies.

Finally, Table 1 displays the numerical statistics obtained from each of the conducted sessions, regarding the number of participants, gender distribution, and an estimation of the time dedicated to each type of coping strategy (individual or organizational level). With the term “other,” the time dedicated to matters unrelated to the research has been accounted for. The time estimation was conducted manually, using content analysis of the recordings to measure the duration dedicated to each topic.

**Table 1:** Numerical statistics obtained from each of the conducted sessions.

Session	Participants, n	Females, n (%)	Males, n (%)	Estimated time in hours dedicated to each type of coping strategies, n (%)		
				Individual	Organization	Other
1	6	4 (67)	2 (33)	2.8 (70)	0.4 (10)	0.8 (20)
2	9	8 (89)	1 (11)	3 (75)	0.6 (15)	0.4 (10)
3	4	1 (25)	3 (75)	3.2 (80)	0.2 (5)	0.6 (15)
4	6	6 (100)	0 (0)	2.4 (60)	1.2 (30)	0.4 (10)
5	4	1 (25)	3 (75)	3.2 (80)	0.6 (15)	0.2 (5)
6	8	3 (38)	5 (62)	2.2 (55)	1.6 (40)	0.2 (5)

### Results 2: Multimedia Psychological Tool for Enhancing Health Professionals' Well-Being

The case study methodology used in this study involved presenting personal experiences that exemplified the various coping strategies identified during the research. The research team carefully selected real-life stories by consensus, choosing those that best represented these strategies and shed light on aspects often overlooked in highly stressful situations.

In the content selection process to develop materials for the case study methodology, the following factors were considered: relevance to the recipients of the training materials to provide valuable insights, clarity of the coping strategy intended to be represented, and presentation of information in a logical, realistic, and structured manner to facilitate learning. We aimed to capture a wide diversity of coping strategies so that the situations under study represented the complexity of the experiences faced and resonated with the personal experiences of different audiences. Preserving the privacy of the narrators was a priority.

The series consists of 4 short podcasts or episodes, ranging from 5 to 10 minutes, with a total duration of 26 minutes and 8 seconds. Titled “BE+ Against COVID, Experiences in Ecuador,” the podcasts are in Spanish and narrated in the first person, with a presenter conducting interviews with HCWs. They are based on focus group information but presented with simplified language and an entertaining aspect for a pleasant listening experience. These podcasts serve as valuable tools to learn and comprehend coping strategies used by HCWs and can be easily adapted to other health care centers facing similar challenges.

The material construction was planned using the protocol in Textbox 2, resulting in the desired outcome. Steps 1 and 2 (objectives and content planning) underwent extensive review by the resilience expert team, while phases 3, 4, 5, and 6 were led by the multimedia engineering subgroup. Peer review was used to ensure the pertinence, clarity, and use of the materials. Triangulation of diverse perspectives was conducted involving experts in the fields of health care, psychology, and multimedia production. Their valuable insights and feedback contributed to refining the content and ensuring the effectiveness of the materials in supporting the well-being of health care professionals.

To enhance accessibility and reach a wider audience, the podcasts have been uploaded to prominent podcast distribution platforms, such as Apple Podcasts, Spotify, Podbean, Amazon Music, and Player FM, among others. The primary webpage hosting the podcast series has been accessible since May 2022 [18].

Multimedia Appendix 1 provides a comprehensive overview of the content covered in each of the 4 episodes.

## Discussion

### Principal Results

The COVID-19 pandemic has placed an enormous strain on health care systems worldwide, forcing medical professionals to make critical decisions in a highly stressful and socially alarmed environment. The impact of the pandemic on health workers has been severe, with many experiencing acute stress reactions, affective pathologies, and psychosomatic reactions [19,20]. Despite the challenges, the professionals in Ecuador, who participated in this study, demonstrated valuable personal resources for coping with the psychological and emotional impact of the pandemic. It is important to identify the factors that contributed to their ability to resist day after day and continue their essential work [21].

Based on the classification results presented in Textboxes 3 and 4, it is evident that emotion-focused ACS comprise individual strategies, such as understanding life complexities, allocating time for relaxation and reflection, and seeking professional counseling or therapy. At the organizational level, strategies arose spontaneously as a solidarity response and not necessarily from institutional planning. Among these social support from coworkers or family members, joining support groups, showing empathy, providing food and beverages, and active listening are effective strategies. Although the pandemic caught everyone off guard, it is a valuable learning experience to improve the institutional role. On the other hand, the problem-focused ACS coping strategies are geared toward taking practical actions to solve the problem. At an individual level, the strategies involve understanding the situation, keeping a daily report, verifying official information, making logical decisions, assessing risks, contributing to the community through solidarity, adhering to protective measures, taking breaks before work, using telemedicine, and maintaining a positive outlook. The organization-level strategies include teamwork, fellowship, team spirit, communication through group chats, and exchanging feedback among colleagues. These strategies help HCWs to remain focused on the task at hand and take effective measures to address the problem. The emotion-focused MACS, or MACS, involve behaviors that do not effectively address the problem and may cause further distress. Isolation is 1 such strategy. Problem-focused MACS include avoiding news related to the pandemic, creating a separate reality, and engaging in risky or extreme activities. Other strategies include pursuing hobbies or pleasurable activities, such as singing or cooking, and engaging in physical exercise or sports. These coping strategies may provide temporary relief but do not address the root cause of the problem and may lead to additional negative consequences.

The results also revealed that individual experiences held greater prominence compared to those related to organizational aspects, both in terms of the time devoted to discussing them (Table 1), where they accounted for the majority of the session time, and in their enumeration, as they were less represented in the classification lists (Textboxes 3 and 4). This might be attributed to the strong emotional intensity present during the group sessions.

This study also shows that the response of health professionals to the pandemic was not only a function of their personality traits but also influenced by the support they received from their organization and the coping strategies they used. As suggested in other studies [22,23], the previous institutional approaches such as work morale, task satisfaction and performance, and leadership styles have usually influenced responsiveness. The role of middle managers was especially important in providing support and guidance to the frontline health workers. They were instrumental in establishing and communicating protocols, ensuring the availability of personal protective equipment, and coordinating resources. This study also highlights the importance of empowering middle managers to support their teams and provide the necessary resources during challenging times [24].

The coping strategies used by the health workers in this study were found to be useful in managing the stress and anxiety associated with the pandemic. These strategies included engaging in hobbies or pleasurable activities, seeking social support, practicing mindfulness and meditation techniques, and engaging in physical exercise. Interestingly, factors such as religion, family, and entertainment like web-based streaming platforms were also found to be common coping mechanisms. By identifying these coping strategies, health care organizations can provide support and resources to their workers to help them manage the stress of their work [25].

The institutional support provided to the health workers during the pandemic was crucial, but it was also found to be insufficient [25,26]. Maintaining the responsiveness and morale of the health workforce became a practical necessity and an objective of all health institutions and systems worldwide, including in Ecuador. For this reason, psychological first aid was organized, and the psychiatry and psychology departments usually offered advice and care to their colleagues [14,27]. However, as noted in other studies, most professionals chose to cope with the situation with their own personal resources and did not demand, to the extent expected, the institutional support provided to them [28]. This study highlights the need for health care organizations to go beyond providing institutional support to recognize the importance of individual resources and provide tools and support to promote individual resilience. Such support can take the form of employee assistance programs, mental health support, peer support resources [11,29], wellness programs [30], and training to help staff manage their personal resources and cope with the stress of their work [31]. In situations such as the pandemic, where social distancing is crucial, digital tools can be a useful way to provide emotional and psychological support [32]. In this way, a psychological aid tool for enhancing the well-being in podcast format allows for quick and agile dissemination.

Another finding of the study was the importance of recovery time for health workers. Health workers demonstrated a clear preference for disconnecting from work at the end of their shift and engaging in activities that helped them recover for the following day. This highlights the importance of managing workload and providing adequate rest periods to avoid burnout.

Overall, this study has provided valuable insights into the coping strategies used by health care professionals during the COVID-19 pandemic in Ecuador. The study highlights the importance of middle managers in providing support and guidance to health workers and the role of individual coping strategies in promoting resilience. The findings of the study suggest that health care organizations should provide both institutional and personal support to promote the resilience of health workers during challenging times.

### Limitations

The classification of coping strategies based on the degree of adaptation has limitations in situations that, due to their nature, exceed the capacity of individuals and systems to respond, as was the case with the COVID-19 pandemic. Engaging in leisure activities as a means of escape constitutes an emotion-focused adaptive strategy, especially in situations where the complete solution to the problem is beyond individual control. However, participants' responses in the study indicated that, in some cases, these strategies were used as a way to avoid the problem. In this work, we are aware of the limitations of this classification based on the degree of adaptation. Despite its inclusion, we advocate for the greater suitability of the classification referring to emotion and problem for analyzing human coping in critical and highly overwhelming situations in which the resolution of the source of distress is beyond individual control.

Another limitation of this study is that the effectiveness of the podcasts has not been evaluated yet. However, we are currently working on analyzing the performance results of the podcasts on each platform to enhance the tool. A significant and necessary future improvement would be the translation of the podcasts into other languages, as the initial version is only available in Spanish.

### Conclusions

The study found that the coping strategies used by health care professionals in Ecuador were categorized into 4 types, which included emotion-focused ACS, problem-focused ACS, emotion-focused MACS, and problem-focused MACS. Through the use of focus groups, health care professionals in Ecuador were able to identify these strategies and share their experiences with others.

This endeavor has led to the development of a novel multimedia support tool, a podcast series titled “BE+ Against COVID: Experiences in Ecuador.” This tool facilitates the widespread dissemination of coping strategies identified in the study, serving as a valuable resource for Spanish-speaking health care workers worldwide. It represents a case study–based approach to train team leaders, empowering them to enhance the resilience capacity and skills of their team members.

The creation of the new support tool in the form of podcasts is a valuable addition to the health care system as it provides HCWs with a resource that they can use to help them cope with the immense health care pressure and social alarm that has arisen as a result of the pandemic. The podcast series provides anonymous testimonials from health care professionals in Ecuador, allowing them to share their experiences and highlight the coping strategies they used to navigate the challenges they faced.

The dissemination of these coping strategies and the creation of the support tool have been critical in promoting the resilience of HCWs in Ecuador and the Spanish-speaking world. As the pandemic continues to affect health care systems around the world, it is essential to identify and implement coping strategies that can help HCWs manage the challenges they face. The “BE+ Against COVID-19” platform and the new podcast series have been successful in achieving this goal and provide a valuable resource for HCWs in need.

## Appendix

Multimedia Appendix 1 Overview of content in podcast episodes.

## Abbreviations

ACS: adaptive coping strategies
HCW: health care worker
MACS: maladaptive coping strategies
WHO: World Health Organization
