# Supplementary material for: Coping Strategies Used by Health Care Workers in Ecuador During the COVID-19 Pandemic: Observational Study to Enhance Resilience and Develop Training Tools
Source: JMIR Hum Factors. 2023 Sep 6;10:e47702. doi: 10.2196/47702 (PMC10512111; doi:10.2196/47702)
Supplement: Multimedia Appendix 1 [file humanfactors_v10i1e47702_app1.docx]

#### Podcast series "BE+ Against COVID, Experiences in Ecuador”

This series titled "Be Positive Against COVID, Experiences in Ecuador" focuses on the coping strategies employed by healthcare professionals in Ecuador during the COVID-19 pandemic. It gathers testimonies from healthcare professionals in Ecuador, narrating their experiences under the pressures of providing care during the pandemic, with a particular emphasis on the coping strategies utilized. This project has been funded by the Miguel Hernández University and the Valencian Government (Conv. GVA-UMH Solcif. 2020/0005).

The language used in this initial distribution of the podcast is Spanish.

To facilitate distribution and broaden the audience, the podcasts have been uploaded to leading podcast distribution platforms, including Apple Podcasts, Spotify, Podbean, Amazon Music, Player Fm, and others.

URL for additional information and audio files:

<https://segundasvictimascovid19.umh.es/p/podcast.html>

The series is comprised of four short podcasts or episodes, ranging from 5 to 10 minutes in length, with a total duration of 26 minutes and 8 seconds. The podcasts are narrated in the first person, with a presenter who conducts interviews with healthcare professionals. These podcasts serve as a valuable tool for learning and understanding coping strategies used by healthcare professionals, and can be easily adapted to other healthcare centers facing similar challenges.

The next subsections will provide a brief overview of the content of each of the four episodes:

#### Chapter 1. Introduction

The inaugural episode serves as an introduction to the series, clarifying its objective, outlining its content, and detailing its four-chapter organization. In the following episodes, a host speaks with different HCWs who narrate their experiences of resilience in first-person and in a simple and understandable manner. In the following sections, we have reproduced some of these narrations (in italics).

#### Chapter 2. Emotion-focused ACS

This audio presentation provides examples of Emotion-focused ACS that were most effective for HCWs in managing the stress associated with the acute phase of the pan-demic. During this period, HCWs were faced with highly emotionally charged situations that required them to effectively manage their emotions. The well-being of healthcare professionals was significantly impacted during the COVID-19 pandemic as a result of a multitude of factors, including the death of patients, the loss of colleagues, resource shortages, uncertainty, and organizational challenges, as well as measures such as social distancing and increased working hours. These circumstances often resulted in feelings of exhaustion, sadness, fear, inadequacy, frustration, and helplessness among health professionals. Despite these challenges, many were motivated by a sense of vocational duty and moral obligation to their role as caregivers, which served as a driving force in their continued efforts on the front lines of the fight against the pandemic.

In this context, the application of Emotion-focused ACS was essential for effectively managing this crisis. Due to the nature of the situation, HCWs were frequently exposed to the stressors associated with the pandemic for prolonged periods of time. As such, strategies that allowed for temporary detachment from the pandemic had a significant positive impact on their mental well-being. These coping mechanisms, such as meditation, seeking social support, therapy, or religious faith, served as effective outlets for alleviating emotional pressure and replenishing resilience, enabling HCWs to sustain their tireless efforts on a daily basis.

Among some of the actions narrated by the HCWs and that have been used as material for this second chapter about Emotion-focused ACS, we highlight the following three HCW storytelling’s, the first one about “Practice mindfulness and meditation techniques”:

‘In my case, I took refuge in meditation. Exercising daily was the way I found to stay calm and do a mental reset so that I could continue going to the hospital, even though I was aware of what I was going to find. (HCW1)’

In this second example, the significance of religious faith in maintaining daily life is discussed:

‘I believe that what helped me to continue was faith. No matter what religion you have, in such a dark time of darkness you need something to help you. We lost three colleagues that you normally saw working side by side and that was very hard. (HCW2)’

And in this final example, the satisfaction of receiving food and drink along with messages of gratitude for one's efforts is discussed:

‘I would say that one of the things that most encouraged me to continue were the donations from people, which were also usually accompanied by positive messages and encouragement. For example, they brought us water, since dehydration was important with the PPE. There was also one day that they brought us dinner, another some muffins... (HCW3)’

#### Chapter 3. Problem-focused ACS

This third chapter addresses the Problem-focused ACS that worked for HCWs to not give up and continue day by day in the fight against COVID-19. These coping strategies involve activities aimed at solving problems or restructuring thoughts that involve changing the situation or its meaning. The information obtained from the focus groups reveals several activities that HCWs implemented to cope with the pandemic. For instance, some HCWs found official sources of information helpful, as it was a time when a lot of fake news was circulating, leading to misinformation and fear:

‘Information from official sources helped me a lot. It was a time when a lot of fake news was circulating, a lot of lies... all it did was misinform people and create more fear than there was. That is why I tried to ignore everything that came to me and only believe what was transmitted by official sources. (HCW4)’

Other HCWs mentioned that being busy with different tasks within the hospital helped them to keep themselves occupied and not slow down. For instance, one HCW stated:

‘I perceived that having to carry out different tasks within the hospital helped us in some way to be busy all the time and not to slow down. For example, I am a clinical psychologist, but during the peaks of the different waves I had to perform other tasks such as delivering death certificates. (HCW5)’

Additionally, group chats played a crucial role in keeping HCWs connected and informed. HCWs shared information and received feedback from their colleagues:

‘I think that something that was very useful at that time were group chats. Through these chats we could send each other information, make a daily report, we shared from laboratory tests to the clinical picture of a patient to be able to discuss how to proceed with that patient. It was very practical to have those chats, which somehow served to get feedback from colleagues. (HCW6)’

The problem-focused ACS mentioned above served as temporary measures to cope with the healthcare pressure generated by the pandemic. These strategies were helpful in enabling HCWs to remain focused on the task at hand and take effective measures to address the problem, ultimately providing quality care to patients.

#### Chapter 4. MACS

In this last episode we have experiences based on maladaptive or non-productive coping strategies (MACS) that helped HCWs not give up and continue going to work during the hardest moments of the COVID-19 pandemic. These types of strategies are based on activities aimed at avoiding the problem or stressful situations and experimenting with wishful thinking, such as fantasizing about alternative realities. MACS can be tempting during times of high stress, but they often do not provide a long-term solution to the problem. It's important to recognize the potential drawbacks of these strategies so that individuals can choose more effective coping methods.

For example, isolating oneself from others may temporarily relieve stress, but it can lead to feelings of loneliness and social disconnection over time:

‘What I resorted to the most at that time was to isolate myself. All I wanted to do was take a shower, get into bed, and close my eyes. I needed to have as my space when leaving the hospital. I was lucky to be able to stay in one of the hotels that enabled health personnel, so that also allowed me to be calmer and rest, since I was not afraid of infecting my relatives. (HCW7)’

Similarly, engaging in extreme activities or hobbies may provide a temporary distraction:

‘Once there were fewer restrictions, I began to do extreme things: skydive, learned to scuba dive, got tattoos, dyed my hair... I did millions of things that I would never have done before in my life and possibly, if it weren't for the pandemic, I wouldn't have dared. (HCW8)’

Also indulging in hobbies or leisure activities can be a healthy way to unwind:

‘I chose to promote my hobbies and thus found a breather. I read more, listened to music... I also really like to sing, and I feel that music helps a lot to release emotions. Other colleagues with whom I have discussed this same topic were inclined to cook. They came home at night and liked to close themselves in the kitchen to prepare their family's food for the next day, or make bread, cakes... It was their way of escape. (HCW9)’

Physical exercise can be a productive way to release tension and improve mental well-being:

‘I started to exercise more. First at home, I did gym exercises, boxing, with the elliptical... Then when the measures were relaxed and it was possible to go jogging, I went every day, I also signed up for swimming... It was a way in which he could release tension and renew energy. (HCW10)’

It is important for HCWs to be aware of these MACS and seek out more effective ways to cope with the stress and pressure of their work.

All the real-life experiences narrated in the podcast series can serve as a reminder to all healthcare professionals of the importance of identifying and using effective coping strategies to maintain their well-being and provide quality care to their patients.


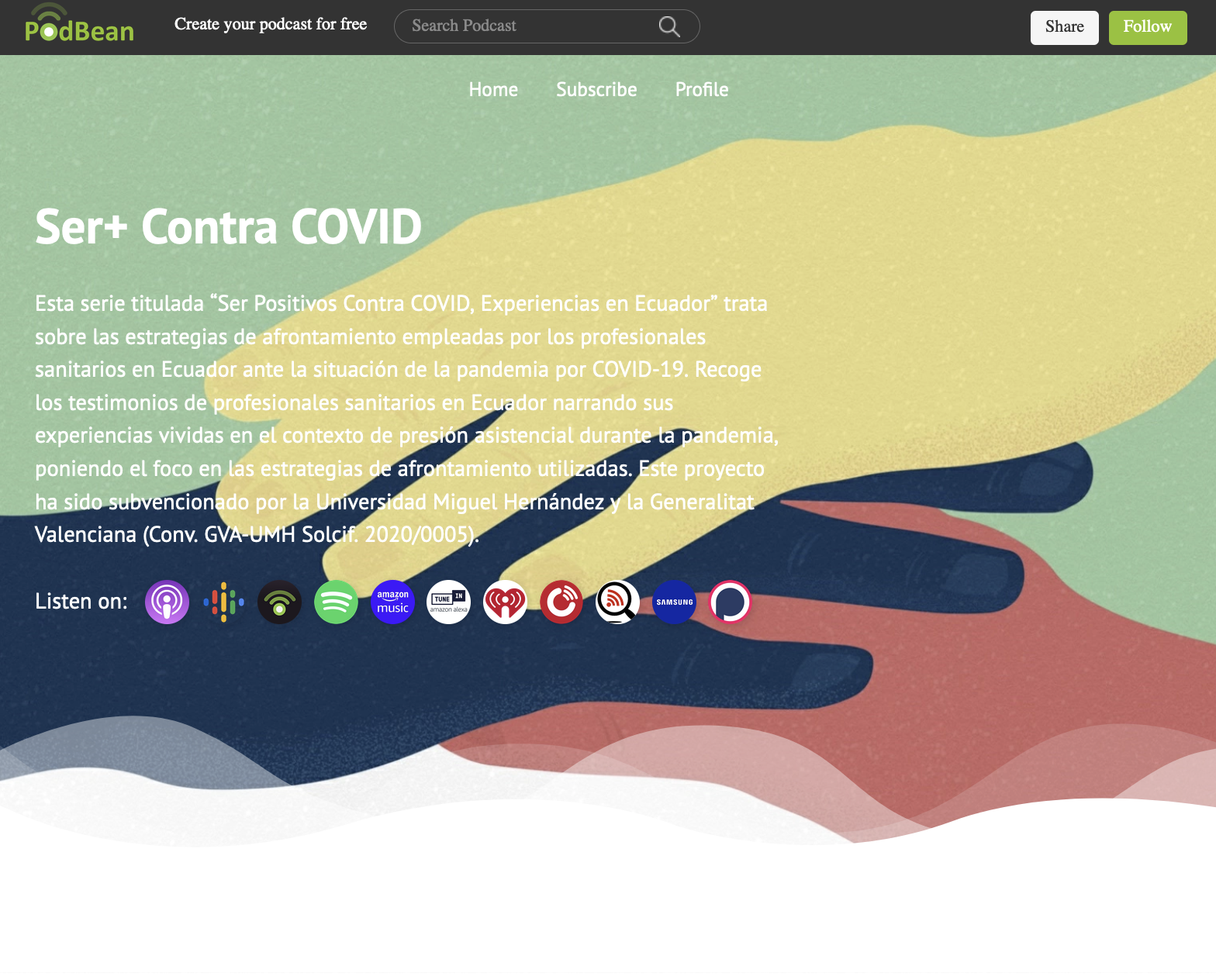


Figure S1. Screenshot from the distribution site of the series podcast in PodBean platform with the links to another platforms (Spotify, Amazon Music, …)


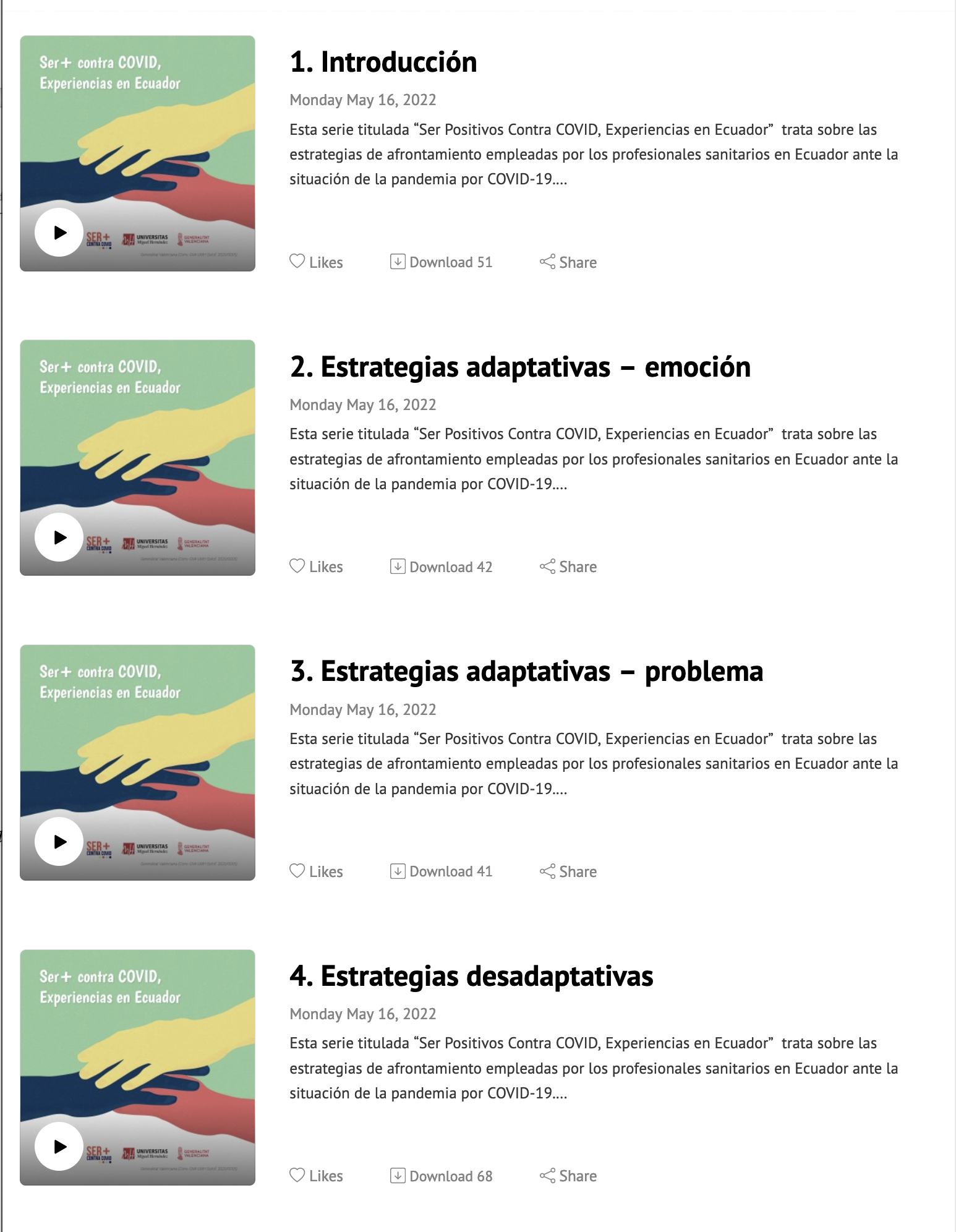


Figure S2. Screenshot with the four podcast audio archives.
